# Effects of footwear and foot strike patterns on patellofemoral joint and Achilles tendon loading in novice runners and experienced runners

**DOI:** 10.3389/fspor.2025.1610514

**Published:** 2025-06-20

**Authors:** Yuxin Li, Yuhang Nie, Xini Zhang, Yaodong Gu

**Affiliations:** ^1^ Faculty of Sport Science, Ningbo University, Ningbo, China; ^2^ Research Academy of Grand Health, Ningbo University, Ningbo, China

**Keywords:** novice runners, foot strike pattern, Achilles tendon, patellofemoral joint, minimalist shoes

## Abstract

**Introduction:**

This study aimed to investigate the differences in the loading characteristics of the patellofemoral joint (PFJ) and the Achilles tendon (AT) between novice and experienced runners across different footwear conditions (conventional vs. minimalist shoes) with rearfoot striking (RFS) and forefoot striking (FFS).

**Methods:**

Eleven male RFS novice runners and experienced runners were randomly asked to run across a force platform at 12 km/h when wearing conventional and minimalist shoes with RFS and FFS, respectively. AT and PFJ loading were estimated from kinematic and kinetic data. The morphological (length and cross-sectional area) of AT *in vivo* were recorded using ultrasound imaging. Three-way ANOVA was used to determine differences in PFJ and AT loading characteristics.

**Results:**

Patellofemoral contact force and AT impulses were significantly greater (*p* < 0.05) in novice runners than in experienced runners, regardless of footwear or the foot strike pattern. Regardless of running level, patellofemoral contact force and PFJ stress were significantly lower in FFS than in RFS, whereas AT force, AT impulse, and peak AT stress were significantly greater in FFS than in RFS. Peak impact loading rates were significantly lower in conventional shoes with RFS than in minimalist shoes (*p* < 0.05).

**Discussion:**

Novice runners experienced a significant increase in PFJ and AT loads during running. In addition, FFS increased the impulse, force, and stress on the AT and decreased the PFJ stress. Therefore, novice runners need to gradually adjust their foot strike pattern according to the loading capacity of different joints to reduce the corresponding injury risk.

## 1 Introduction

Running is one of the most popular sports in the world, and as an increasing number of people enjoy running, running injuries are inevitable. The patellofemoral joint (PFJ) and Achilles tendon (AT) are the most common injury sites in running, with the incidence of patellofemoral pain syndrome and Achilles tendinopathy accounting for 15.6% and 9.5%, respectively, of all running injuries (1). AT or PFJ injuries can lead to pain and restriction of activity for runners, reducing their quality of life, suggesting that Achilles tendinopathy and patellofemoral pain syndrome are important sociomedical issues (2). Overuse injuries were a major contributor to patellofemoral pain syndrome and Achilles tendinopathy. During running, prolonged high loads and repetitive cyclic loading on the PFJ and AT lead to microinjuries, and insufficient recovery time exacerbates these, ultimately resulting in macroscopic damage (3). Although no clear data currently exist regarding differences in PFJ and AT injury incidence rates between novice and experienced runners, research has shown that novice runners are 17.8% more likely to sustain running injuries than recreational runners (4). The rate of medical attention required by novice runners postinjury was 36.8%, which was significantly higher than the 29.2% reported in recreational runners (5). Given the high incidence of running injuries and the associated healthcare costs, strategies to reduce the risk of running injuries must be investigated, and effective prevention programs, particularly for novice runners, must be developed.

Previous studies have investigated that the high incidence of running-related injuries of the PFJ and AT are related to foot strike patterns or footwear conditions. Most studies have shown that, compared to wearing minimalist shoes or adopting a forefoot striking (FFS) pattern, wearing cushioned running shoes or adopting a rearfoot striking (RFS) pattern increases knee flexion angles and knee moments, which, in turn, raises the load on the PFJ and increases the risk of patellofemoral pain syndrome (6–8). Conversely, ankle plantar flexion moments, AT forces and loading rates are decreased, potentially lowering the risk of AT injury (9–12). However, while the effects of different footwear types or foot strike patterns on PFJ and AT loading have been explored, these studies often fail to account for the lower extremities as an integrated kinetic chain. Palmitier et al. (13) pointed out that the hip, knee, and ankle act as an integrated kinetic chain within the lower extremity. Within this chain, the PFJ is a key structure within the knee joint system, whereas the AT is equally critical to the ankle-calf complex. Consistently, our recent research showed that the FFS pattern decreased PFJ loading but increased mechanical demand on the AT, whereas the RFS pattern produced the opposite effect (14). These findings underscored that the PFJ and AT were not isolated structures, but components of a cohesive system in which forces and loading patterns influenced each other (3, 15, 16). Most previous studies have focused exclusively on either the PFJ or the AT, neglecting the interrelationship within the entire kinetic chain of the lower limb. This approach has resulted in isolated conclusions, making it difficult to directly compare PFJ and AT loading across studies and limiting our understanding of how these injuries occur within the context of the whole lower limb.

Furthermore, the loading of the PFJ and AT in novice runners has yet to be fully investigated, as current studies have focused mainly on trained athletes or patients with patellofemoral pain syndrome and Achilles tendinopathy (15, 17). Indeed, significant differences in knee joint and ankle joint mechanics have been observed between novice and experienced runners. Kinematic analyses revealed that novice runners had significantly greater ankle inversion angles and range of motion (ROM), whereas experienced runners exhibited greater knee flexion angles (18). Kinetically, peak ankle inversion moments and peak internal rotation moments were higher in novice runners than in experienced runners (18–20). In addition, higher vertical instantaneous loading rates were observed in novice runners compared to experienced runners (18). These differences in joint mechanics could potentially influence the loading patterns on the PFJ and AT. Given that novice runners are more susceptible to running-related injuries, understanding the unique PFJ and AT characteristics under different foot strike patterns and footwear conditions is essential for developing targeted prevention strategies.

This study aimed to investigate the differences in loading characteristics on the PFJ and AT between novice and experienced runners habituated to RFS across different footwear (conventional shoes and minimalist shoes) and foot strike patterns (RFS and FFS). We hypothesised that (1) PFJ and AT loads would be significantly greater in novice runners than experienced runners, regardless of the foot strike pattern or footwear; (2) novice and experienced runners could immediately reduce the PFJ load but increase the AT load by running with an FFS in minimalist shoes compared with conventional shoes; and (3) there would be significant differences in spatiotemporal gait parameters and joint mechanics between novice and experienced runners across different footwear and foot strike patterns.

## 2 Methods

### 2.1 Participants

The sample size was estimated via G*power software (3.0.1, Univ. Kiel, Kiel, Germany) via *a priori* power analysis of the data published by Van Hooren et al. (3), who investigated the effects of speed, surface gradient and cadence on lower limb loading and a partial *η*^2^ of 0.56. The results revealed that the minimum sample size required for three-way ANOVA was 12. Considering the possibility of attrition, a total of 22 healthy male runners were recruited for this study, including 11 experienced runners (age: 33.7 ± 8.7 years, height: 175.3 ± 4.5 cm, body mass: 69.6 ± 7.0 kg, weekly running distance: 37.8 ± 16.4 km, running experience: 5.3 ± 2.3 years) and 11 novice runners (age: 20.5 ± 1.9 years, height: 176.9 ± 4.8 cm, body mass: 73.5 ± 8.6 kg, weekly running distance: 2.8 ± 1.2 km, running experience: 0.4 ± 0.1 years), who habitually wore conventional shoes with RFS and exhibited a dominant right leg. Experienced runners were required to have a minimum weekly running distance of 20 km over the last 4 weeks. Novice runners are required to train irregularly or regularly for less than 6 months, run fewer than two to three times per week for less than 10 km and/or less than 45 min per run but can complete one 30-minute and/or 5-km run at a pace of their choice (21, 22). All the participants had no lower limb injuries within the past year, no joint laxity and no prior experience with barefoot or minimalist shoe running. Additionally, they were instructed not to consume caffeine or alcohol 2 h before the experiment and to avoid intense or exhaustive exercise 24 h prior. The study procedures complied with the Declaration of Helsinki. The study was approved by the Ethics Committee of Ningbo University (No. TY2023002). All participants signed written informed consent before the measurements were taken.

### 2.2 Experimental procedures

An ultrasonography test was first performed to obtain the participant's resting AT length via an ultrasonographer (QSONO Q6, Wuhan, China). At the beginning of the test, the participant was asked to assume a prone position with the knee flexed at 10°. A triangular foam pad was placed in front of (under) the ankle joint with the feet resting in a relaxed position. A goniometer was used to position the ankle at 10° of plantar flexion by adjusting the foam pad. Once the coupling agent is applied to the probe and the probe is placed vertically at the insertion point of the AT, the researcher can insert a 21-gauge needle between the ultrasound probe and the skin surface, which serves as a marking projection in the ultrasound image. The intersection point between the needle and the ultrasound probe was then marked on the skin with a pen. The same operation marked the point where the medial head of the gastrocnemius muscle and the AT meet. The distance between these two points was then measured via a soft ruler, which represents the length of the AT (23). The ultrasound probe was then positioned perpendicular to the skin surface, and the cross-sectional area of the AT was collected at the level corresponding to the medial and lateral ankles.

**Figure 1:**
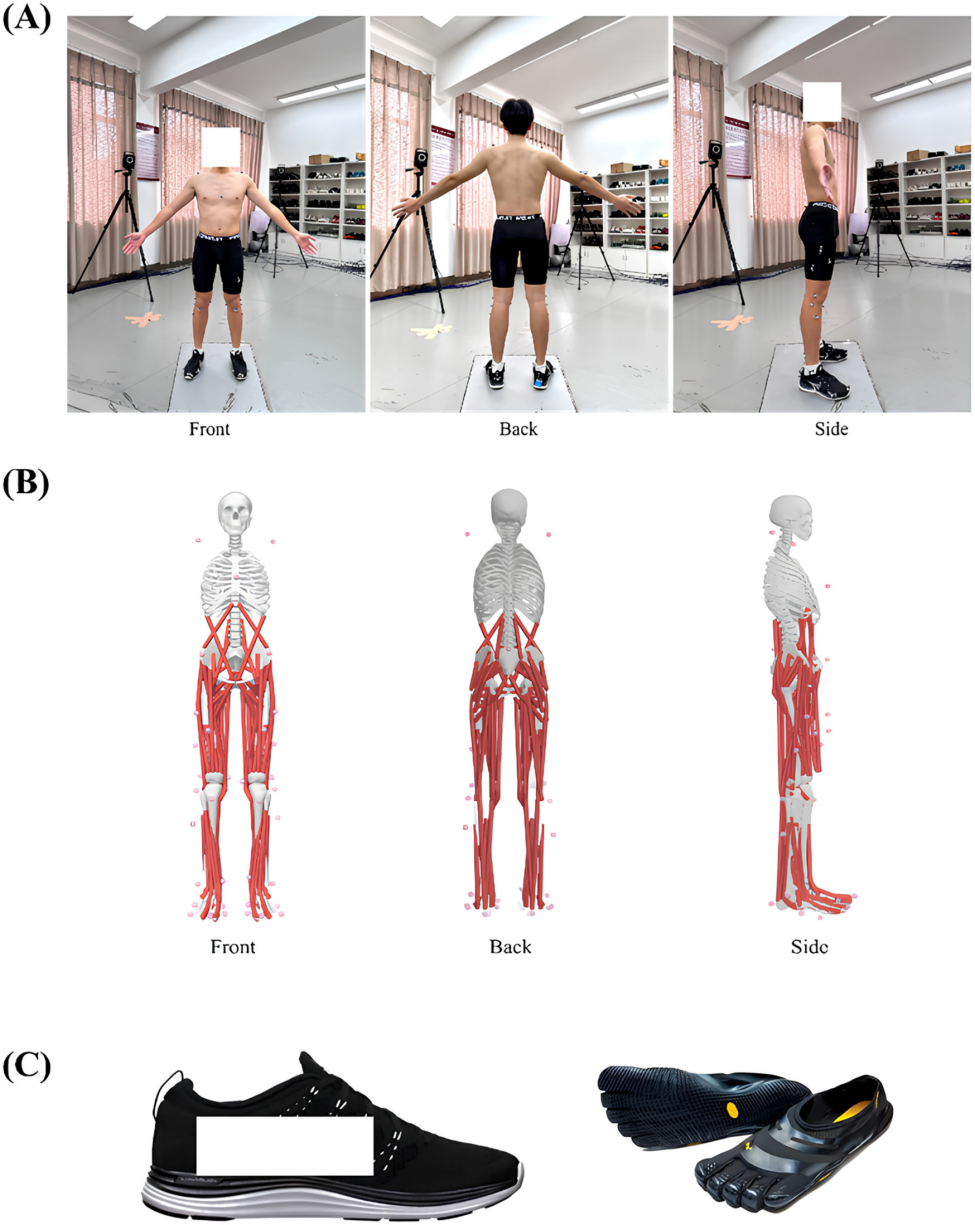
Positioning of the 38 reflective markers and experimental shoes. **(A)** Positioning of the 38 reflective maker points on the participant's body. **(B)** Positioning of the 38 reflective marker points on the 3D models of bones and muscles. **(C)** Conventional shoes (left) and minimalist shoes (right) used for the experiment.

The participants were instructed to change into the experimental vest, shorts, socks and randomly selected running shoes, namely, conventional shoes (Lunarlon foam midsole, 10.8 mm heel-to-toe drop, average weight 232.46 g, sizes 41–45) and minimalist shoes (3 mm rubber outsole, no midsole, 0 mm heel-to-toe drop, Figure 1C). Before the experiment, participants were given 10 min to adapt to the different footwear and foot strike patterns (24). After a 5-minute warm-up at a self-selected pace, 38 reflective markers with a diameter of 14.0 mm were placed on the participants (Figures 1A,B). After the static model was collected, the participants were asked to run across the 3D force platform at a speed of 12 km/h (25) via their habitual foot strike patterns (i.e., RFS). Participants were instructed to maintain their natural running gait while ensuring that the right foot made full contact with the force platform during the stance phase. A trial was deemed valid only if the running speed remained within ±5% of the prescribed 12 km/h. Each participant completed five successful data collections, with a break of at least 1 min between different test conditions. The participants were subsequently guided to transition to an FFS pattern. Through recording and observing the participants' foot strike patterns in real-time using high speed 3D infrared cameras (Vicon Metrics, Ltd., Oxford, UK, 200 Hz), while the vertical ground reaction force (GRF) trace was simultaneously inspected for the single peak profile that typifies FFS. In post-processing, foot strike angle (FSA) was further calculated to determine whether participants were RFS or FFS. Footwear was chosen randomly, and after completing the first pair of shoe tests, the participants were asked to change their shoes and repeat the experiment. The running speed was recorded via a browser timing device (Brower Timing System, Draper, UT, USA) on a 20 m runway. During this phase, both the reflective marker trajectories and GRFs were recorded simultaneously. The reflective marker trajectories were captured via an eight-camera infrared 3D motion capture system at a sampling rate of 200 Hz to obtain kinematic data (18, 25, 26). The GRF parameters during the running stance phase were synchronised and collected via a 600 mm × 400 mm 3D force platform (AMTI, Watertown, MA, USA) at a sampling rate of 1,000 Hz.

### 2.3 Data processing

**Figure 2:**
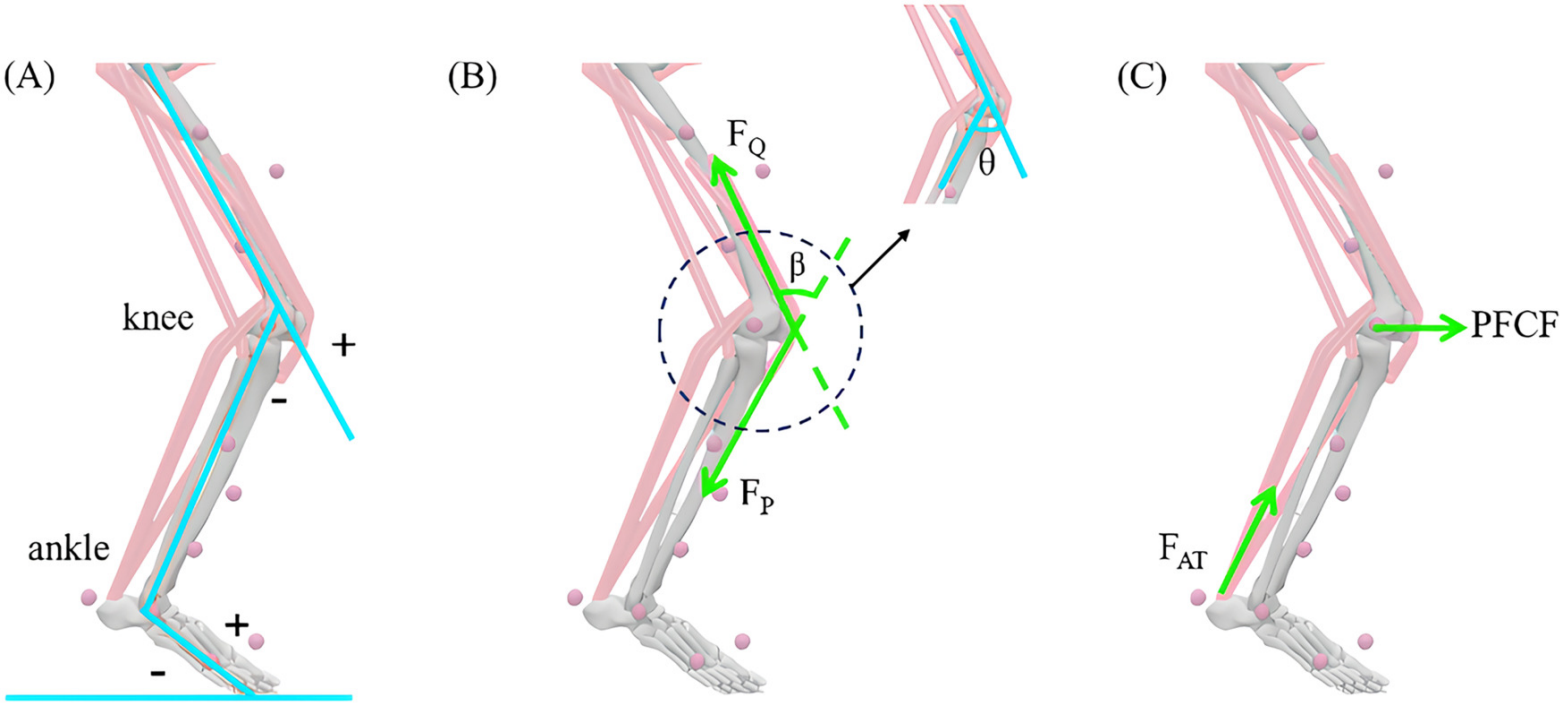
Sagittal plane angles of the three lower limb joints in the stance phase and free-body diagram of the patellofemoral joint. **(A)** The joint angle of the lower limb's three joints in static standing is 0°, “+”: knee extension and ankle dorsiflexion; “−”: knee flexion and ankle plantar flexion. **(B)** *F*_Q_ refers to the quadriceps muscle strength, *F*_P_ represents the patellar ligament tension line, *β* represents the angle between the quadriceps muscle line and the patellar ligament tension line, and *θ* represents the knee flexion angle. **(C)** PFCF denotes the patellofemoral contact force, and *F*_AT_ denotes the Achilles tendon force.

The participants in this study had the right leg as the dominant leg, so the reported variables were calculated from the right leg data. The reflective marker trajectories and GRFs were imported into MATLAB (R2022a, MathWorks, Natick, MA, USA) for data extraction, filtering, and format conversion. Kinematic and kinetic data were filtered using fourth-order Butterworth low-pass filters with cutoff frequencies of 10 and 20 Hz (27). The processed data were subsequently subjected to static model scaling (Model 2392), inverse kinematics and inverse dynamics calculations via OpenSim (v4.4, Stanford University, Stanford, CA, USA). The analysis focused on the stance phase, from initial foot contact to toe-off, defined as when the GRF exceeded 30 N (28). The angles of the three joints of the lower limb in the sagittal plane were calculated using Euler angles, with neutral positions at 0°. Positive values indicated knee extension and ankle dorsiflexion, and negative values denoted knee flexion and ankle plantar flexion (Figure 2A). The ROM for the knee and ankle was calculated from the maximum and minimum angles during the stance phase. Knee extension and ankle plantar flexion moments were determined via inverse dynamics and normalized to body weight (BW). The parameters related to the stance phase were time normalised (0%–100%).

The FSA was defined as the angle between the line connecting the heel to the first toe and the ground and was calculated by subtracting the angle between the foot and ground at contact (GRF > 30 N) from the angle at rest. FFS was defined as an FSA < −1.6°, midfoot striking as an FSA between −1.6° and 8° and RFS as an FSA > 8° (29). The step frequency was the number of times the foot contacted the ground per minute. The contact time was the time from when the same-side leg contacted the ground to toe-off. Flight time was the time from when the same-side leg toe-off to when it contacted the ground again. The step length was the horizontal distance between the point of contact of one foot and the point of contact of the opposite foot during running. The duty factor is the proportion of foot contact time in a gait cycle to the entire gait cycle time (30).

The effective arm of the quadriceps force (*L*_A_, Equation 1) was a function of the sagittal knee angle (*θ*_i_,°, Figure 2B) and expressed as follows (31):

(1) $$L_{A}=\{\begin{array}{c} 0.036\theta_{i}+3.0(0^{\circ }\leq \theta_{i}<30^{\circ })\\ -0.043\theta_{i}+5.4(30^{\circ }\leq \theta_{i}<60^{\circ })\\ -0.027\theta_{i}+4.3(60^{\circ }\leq \theta_{i}<90^{\circ })\\ 2.0(90^{\circ }\leq \theta_{i}) \end{array}$$

The quadriceps force (*F*_Q_, N, Equation 2, Figure 2B) was calculated as follows (32):

(2) $$F_{Q}(\theta_{i})=M_{\mathrm{EXT}}(\theta_{i})\;/L_{A}(\theta_{i})$$

where *M*_EXT_ (N·m) is the knee extension moment.

The patellofemoral contact force (PFCF, N, Equation 3, Figure 2C) was calculated as follows (32):

(3) $$\mathrm{PFCF}=2F_{Q}\sin(\beta \;\times \pi /360)$$

where *β* (°) is the angle between the quadriceps line and the patellar ligament (Figure 2B) and where *β* = 30.46 + 0.53 (*θ*_i_).

The patellofemoral contact area (PFCA, mm^2^, Equation 4) is a function of the sagittal knee angle and is expressed as follows (31):

(4) $$\mathrm{PFCA}(\theta_{i})=0.0781\theta_{i}^{2}+0.6763\theta_{i}+151.75$$

where PFCA represents the contact area between the patella and the femur.

The patellofemoral joint stress (PFJS, MPa, Equation 5) was calculated as follows:

(5) $$\mathrm{PFJS}=\mathrm{PFCF}/\mathrm{PFCA}\;(\theta_{i})$$

The peak AT force, which was the peak plantar flexion force, was obtained by dividing the peak plantar flexion moment by the AT force arm, which was based on the polynomial algorithm used by Lyght et al. (33) for calculating the muscular force arm of the calf triceps muscle and was obtained from the *in vivo* AT imaging data of Rugg et al. (34) and normalised for weight. In the AT force–time curve, the peak instantaneous loading rate (ILR) and average loading rate (ALR) of the AT force were determined via the instantaneous and average slopes from the initial contact to the peak force, respectively. The AT force impulse was obtained from the integral of the AT force during the stance phase. The peak AT stress was obtained by dividing the peak AT force by the AT cross-sectional area; an ultrasonic imager was used to obtain the AT cross-sectional area, and the AT cross-sectional area was determined via ImageJ software (NIH, USA). The intraclass correlation coefficients for the intra and interobserver reliabilities, which were from our pilot testing, were good to excellent (ICC = 0.895–0.996) for cross-sectional area. These analyses were performed by previous research (26).

### 2.4 Statistical analysis

All the data are expressed as the means ± standard deviations and were analysed via SPSS statistical software (SPSS v22.0, IBM, Armonk, USA). The obtained parameter values were assessed for a normal distribution via the Shapiro–Wilk test. Three-way ANOVA (running leve × footwear × foot strike patterns) was used to determine differences in spatiotemporal gait characteristics and PFJ and AT loading characteristics. A significant interaction was identified via ANOVA, and differences across running level, footwear or foot strike patterns were quantified via Bonferroni's *post hoc* multiple comparison test. The effect sizes were expressed as Cohen's *d*, where *d* < 0.19 was a small effect, *d* = 0.20–0.79 was a medium effect and *d* > 0.8 was a large effect. The significance level was set at 0.05.

## 3 Results

A significant three-way interaction effect amongst different running levels, footwear and foot strike patterns was observed for the FSA (*p* < 0.05, Table 1). *Post hoc* analyses revealed that novice runners had a significantly smaller FSA when wearing minimalist shoes with FFS than did experienced runners. Moreover, runners at equivalent levels presented significantly lower FSAs when running with FFS than when running with RFS, regardless of footwear. A significant main effect of running level was observed on spatiotemporal gait characteristics (*p* < 0.05), with novice runners showing significantly greater step frequency, contact time and flight time and lower duty factor and step length than experienced runners did.

**Table 1:** Effects of different running levels, footwears and foot strike patterns on spatiotemporal gait characteristics (mean ± SD).

Variables			FSA (°)	SF (step/min)	SL (m)	CT (ms)	FT (ms)	DF (%)
C	FFS	NR	−9.3 ± 5.3	179.9 ± 14.2	1.1 ± 0.1	231.6 ± 15.7	430.5 ± 42.4	35.1 ± 2.5
		ER	−5.2 ± 2.6	175.4 ± 12.2	1.2 ± 0.4	212.7 ± 12.3	219.8 ± 157.6	54.1 ± 15.4
	RFS	NR	10.1 ± 3.3^A^	189.5 ± 15.2	1.1 ± 0.1	228.8 ± 21.3	419.7 ± 21.8	35.3 ± 2.4
		ER	13.8 ± 2.9^A^	170.4 ± 9.4	1.2 ± 0.5	224.8 ± 19.4	200.8 ± 163.5	58.5 ± 16.3
M	FFS	NR	−10.7 ± 4.9	192.1 ± 21.8	1.1 ± 0.1	221.4 ± 28.0	414.2 ± 43.0	34.9 ± 4.1
		ER	−3.6 ± 1.7^B^	174.8 ± 12.9	1.2 ± 0.4	208.9 ± 15.6	223.2 ± 165.9	53.6 ± 16.4
	RFS	NR	10.6 ± 3.1^A^	187.3 ± 13.8	1.1 ± 0.1	226.7 ± 15.9	415.8 ± 31.2	35.3 ± 2.5
		ER	10.4 ± 2.5^A^	178.4 ± 16.7	1.09 ± 0.4	213.5 ± 11.3	178.9 ± 146.7	59.2 ± 14.1
*p*-value (*η*^2^)		RL	<0.001 (0.235)	<0.001 (0.161)	<0.001 (0.353)	0.002 (0.110)	<0.001 (0.489)	<0.001 (0.496)
		FW	0.384 (0.009)	0.176 (0.023)	0.224 (0.018)	0.081 (0.038)	0.695 (0.002)	0.992 (0.000001)
		FSP	<0.001 (0.886)	0.797 (0.001)	0.735 (0.001)	0.219 (0.019)	0.463 (0.007)	0.271 (0.015)
		RL*FW	0.724 (0.002)	0.843 (0.0004)	0.790 (0.001)	0.865 (0.0004)	0.985 (0.000004)	0.971 (0.00002)
		RL*FSP	0.013 (0.075)	0.630 (0.003)	0.507 (0.006)	0.363 (0.010)	0.528 (0.004)	0.331 (0.012)
		FW*FSP	0.301 (0.013)	0.651 (0.003)	0.655 (0.003)	0.968 (0.00002)	0.896 (0.0002)	0.880 (0.0003)
		RL*FW*FSP	0.022 (0.064)	0.075 (0.039)	0.092 (0.035)	0.313 (0.013)	0.702 (0.002)	0.927 (0.0001)

C, conventional shoes; M, minimalist shoes; FFS, forefoot striking; RFS, rearfoot striking; NR, novice runners; ER, experienced runners; FSA, foot strike angle; FSA < −1.6° was FFS; FSA > 8° was RFS; SF, step frequency; SL, stride length; CT, contact time; FT, flight time; DF, duty factor; RL, running level; FW, footwear; FSP, foot strike patterns.

^A^ Significant difference between FFS and RFS at the same running level and footwear, *p* < 0.05.

^B^ Significant difference between NR and ER for the same footwear and foot strike patterns, *p* < 0.05.

**Figure 3:**
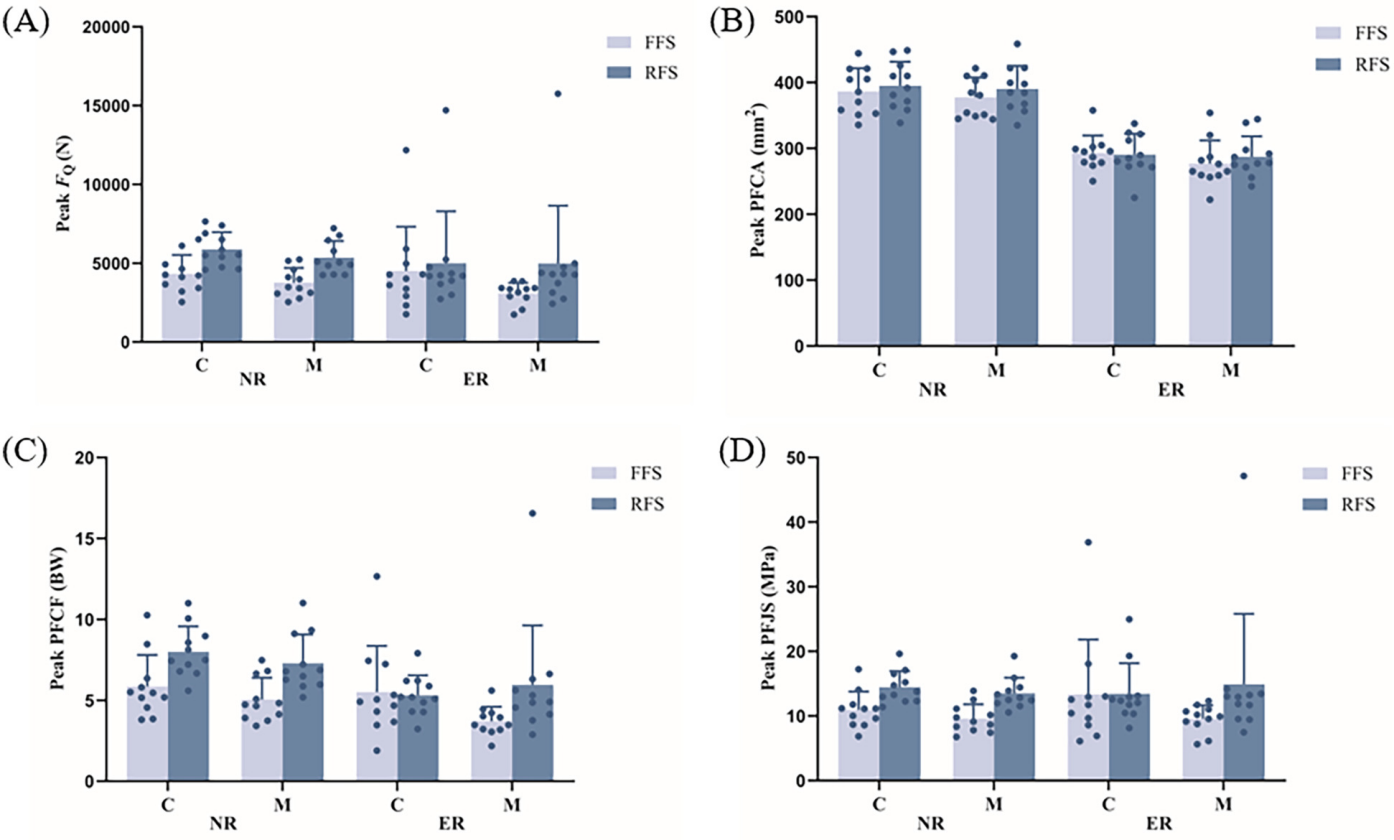
Effects of different running level, footwear and foot strike patterns on peak quadriceps force (*F*_Q_, **A**), peak patellofemoral contact area (PFCA, **B**), peak patellofemoral contact force (PFCF, **C**), and peak patellofemoral joint stress (PFJS, **D**). C, conventional shoes; M, minimalist shoes; FFS, forefoot striking; RFS, rearfoot striking; NR, novice runners; ER, experienced runners.

No interaction was detected between the kinematic and kinetic parameters of the knee joint or the PFJ (*p* > 0.05, Table 2). Running level and foot strike pattern had significant effects on the knee joint angle at contact, knee peak extension angle and peak PFCF (*p* < 0.05, Figure 3C). The knee joint angle at contact and the knee peak extension angle were significantly greater in novice runners and FFS than in experienced runners and RFS. Moreover, the peak PFCF was significantly greater in novice runners than in experienced runners, whereas the peak PFCF was significantly lower with FFS than with RFS, regardless of running level or footwear. Running level had significant effects on the peak knee flexion angle and peak PFCA (*p* < 0.05, Figure 3B). In particular, the knee peak extension angle and peak PFCA of novice runners were significantly greater than those of experienced runners. Significant main effects of running level, footwear and foot strike patterns were shown on knee joint ROM (*p* < 0.05). Additionally, knee joint ROM was significantly greater in novice runners, in conventional shoes and during RFS than in experienced runners, minimalist shoes and FFS. Foot strike patterns had a significant main effect on the peak knee extension moment, peak *F*_Q_ (Figure 3A) and peak PFJS (*p* < 0.05, Figure 3D). Running with an FFS resulted in a significantly lower knee extension moment, peak *F*_Q_ and PFJS than running with an RFS.

**Table 2:** Effects of different running levels, footwears and foot strike patterns on knee joint sagittal plane mechanical parameters and the characteristics of patellofemoral joint loading (mean ± SD).

Variables			Knee joint					PFJ			
			*θ*_contact_ (°)	*θ*_FL-peak_ (°)	*θ*_EX-peak_ (°)	*θ*_ROM_ (°)	*M*_EX-peak_ (Nm/kg)	Peak *F*_Q_ (N)	Peak PFCA (mm^2^)	Peak PFCF (BW)	Peak PFJS (MPa）
C	FFS	NR	32.0 ± 5.4	50.6 ± 4.1	25.53 ± 3.46	25.0 ± 5.0	1.9 ± 0.5	4,330.6 ± 1,191.5	386.4 ± 35.2	5.9 ± 1.9	10.9 ± 2.8
		ER	20.1 ± 5.2	38.2 ± 3.9	20.09 ± 5.16	18.2 ± 4.1	2.4 ± 1.3	4,511.9 ± 2,796.5	293.0 ± 26.7	5.5 ± 2.9	13.4 ± 8.5
	RFS	NR	24.4 ± 6.5	51.5 ± 4.2	22.79 ± 4.42	28.1 ± 3.4	2.6 ± 0.4	5,881.3 ± 1,090.8	395.1 ± 36.3	8.0 ± 1.6	14.5 ± 2.5
		ER	15.2 ± 5.3	38.0 ± 5.1	15.16 ± 5.32	22.8 ± 3.9	2.3 ± 0.6	4,996.5 ± 3,296.2	290.6 ± 31.7	5.3 ± 1.2	13.5 ± 4.7
M	FFS	NR	32.9 ± 4.8	49.5 ± 3.6	26.27 ± 2.40	23.2 ± 4.2	1.7 ± 0.4	3,770.5 ± 922.9	377.6 ± 30.0	5.0 ± 1.4	9.6 ± 2.2
		ER	18.8 ± 6.6	34.3 ± 6.6	18.79 ± 6.57	16.8 ± 4.4	1.6 ± 0.4	3,086.4 ± 674.7	277.0 ± 35.0	3.7 ± 0.9	9.6 ± 2.1
	RFS	NR	29.0 ± 5.9	50.9 ± 4.1	24.34 ± 3.63	26.6 ± 3.0	2.4 ± 0.5	5,353.2 ± 1,050.7	390.2 ± 35.1	7.3 ± 1.8	13.5 ± 2.4
		ER	19.4 ± 4.9	37.3 ± 4.7	19.44 ± 4.90	17.9 ± 3.0	2.6 ± 1.6	4,982.4 ± 3,666.9	287.3 ± 31.0	6.0 ± 3.7	14.9 ± 10.9
*p*-value (*η*^2^)	RL		<0.001 (0.524)	<0.001 (0.708)	<0.001 (0.341)	<0.001 (0.464)	0.581 (0.004)	0.342 (0.011)	<0.001 (0.721)	0.002 (0.112)	0.559 (0.004)
	FW		0.082 (0.037)	0.116 (0.031)	0.186 (0.022)	0.003 (0.105)	0.232 (0.018)	0.173 (0.023)	0.241 (0.017)	0.137 (0.027)	0.326 (0.012)
	FSP		0.001 (0.121)	0.198 (0.021)	0.027 (0.060)	<0.001 (0.154)	0.003 (0.105)	0.004 (0.101)	0.300 (0.013)	<0.001 (0.139)	0.007 (0.086)
	RL*FW		0.609 (0.003)	0.451 (0.007)	0.862 (0.0004)	0.472 (0.006)	0.994 (0.0001)	0.849 (0.0004)	0.842 (0.001)	0.835 (0.001)	0.989 (0.000002)
	RL*FSP		0.129 (0.029)	0.933 (0.00009)	0.921 (0.0001)	0.700 (0.002)	0.537 (0.005)	0.683 (0.002)	0.634 (0.003)	0.195 (0.021)	0.674 (0.002)
	FW*FSP		0.057 (0.044)	0.344 (0.011)	0.111 (0.031)	0.251 (0.016)	0.132 (0.028)	0.435 (0.008)	0.553 (0.004)	0.156 (0.025)	0.233 (0.018)
	RL*FW*FSP		0.690 (0.002)	0.480 (0.006)	0.234 (0.018)	0.335 (0.012)	0.158 (0.025)	0.435 (0.007)	0.753 (0.001)	0.194 (0.021)	0.303 (0.013)

C, conventional shoes; M, minimalist shoes; FFS, forefoot striking; RFS, rearfoot striking; NR, novice runners; ER, experienced runners; RL, running level; FW, footwear; FSP, foot strike patterns; *θ*_contact_, joint angle at contact; *θ*_FL-peak_, peak flexion angle; *θ*_EX-peak_, peak extension angle; *θ*_ROM_, range of motion; *M*_EX-peak_, peak extension moment; PFJ, patellofemoral joint; *F*_Q_, quadriceps force; PFCA, patellofemoral contact area; PFCF, patellofemoral contact force; PFJS, patellofemoral joint stress.

**Figure 4:**
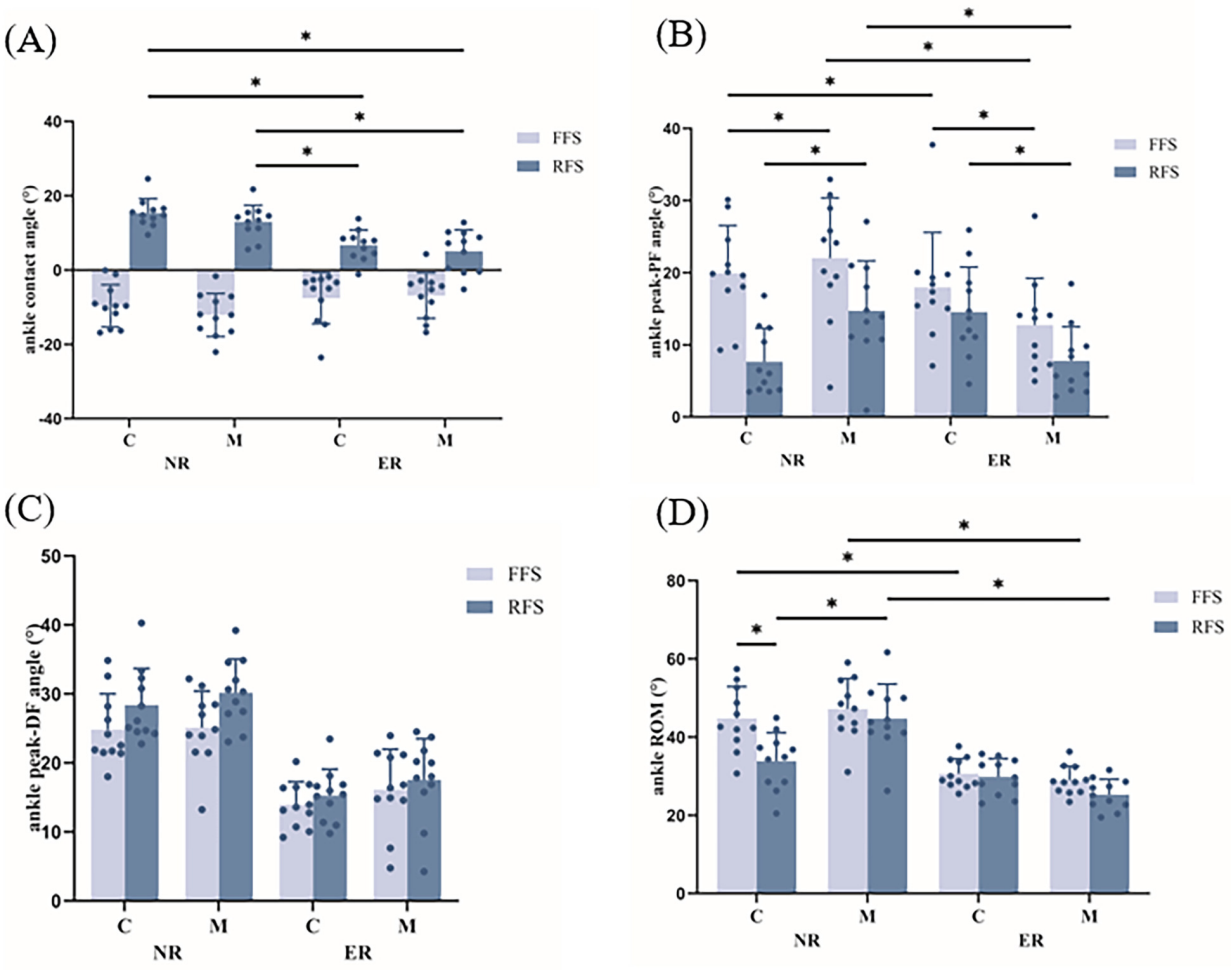
Effects of different running level, footwear and foot strike patterns on ankle contact angle **(A)**, ankle peak plantarflexion angle (peak-PF, **B**), ankle peak dorsiflexion angle (peak-DF, **C**) and ankle range of motion (ROM, **D**), *p < 0.05. C, conventional shoes; M, minimalist shoes; FFS, forefoot striking; RFS, rearfoot striking; NR, novice runners; ER, experienced runners.

A significant three-way interaction effect amongst running level, footwear and foot strike pattern was observed for ankle joint ROM (*p* < 0.05, Figure 4D). *Post hoc* analyses revealed that novice runners had significantly greater ankle joint ROM than experienced runners did when running with FFS in conventional shoes, regardless of whether they were transitioning to FFS or RFS in minimalist shoes. Furthermore, ankle joint ROM was significantly lower in novice runners wearing conventional shoes than in those wearing minimalist shoes when running with RFS. Additionally, ankle joint ROM was significantly greater in novice runners when running in conventional shoes with FFS than with RFS. A significant interaction effect between running level and foot strike pattern was observed for the ankle joint angle at contact (Figure 4A) and the peak ankle plantar flexion angle (*p* < 0.05, Figure 4B). *Post hoc* analyses revealed that the ankle joint angle at contact was significantly greater in novice runners with RFS than in experienced runners with the same footwear. The ankle peak plantar flexion angle was significantly greater in novice runners when running with FFS than in experienced runners in the same footwear. Furthermore, the ankle joint angle at contact was significantly smaller and the ankle peak plantar flexion angle was significantly greater in runners at the same running level and footwear with FFS than in those with RFS. A significant interaction effect between running level and footwear was observed for the ankle peak plantar flexion angle (*p* < 0.05, Figure 4B). *Post hoc* analyses revealed that regardless of the foot strike pattern, the ankle peak plantar flexion angle was significantly greater in novice runners than in experienced runners when running in minimalist shoes and significantly smaller in novice runners when wearing conventional shoes than minimalist shoes. Conversely, experienced runners presented significantly greater ankle peak plantar flexion angles with conventional shoes than with minimalist shoes, regardless of the foot strike pattern. Running levels and foot strike patterns had significant effects on the peak ankle dorsiflexion angle (Figure 4C) and peak plantar flexion moment (*p* < 0.05, Table 3). The ankle peak dorsiflexion angle and peak plantar flexion moment were significantly greater in novice runners than in experienced runners. Additionally, the peak plantar flexion moment was greater when running with an FFS than with a RFS, whereas the peak ankle dorsiflexion angle was smaller with FFS than with RFS.

**Table 3:** Effects of different running levels, footwears and foot strike patterns on ankle joint sagittal plane parameters and the characteristics of Achilles tendon loading (mean ± SD).

Variables			Ankle joint					AT				
			*θ*_contact_ (°)	*θ*_DF-peak_ (°)	*θ*_PF-peak_ (°)	*θ*_ROM_ (°)	*M*_PF-peak_ (Nm/kg)	Peak force (BW)	Peak ILR (BW/s)	Peak ALR (BW/s)	Impulse (BW·s)	Peak stress (MPa)
C	FFS	NR	−9.6 ± 5.7	24.8 ± 5.2	19.9 ± 6.6	44.7 ± 8.1	3.7 ± 0.6	6.9 ± 1.1	127.8 ± 37.8	58.1 ± 11.7	0.9 ± 0.2	78.2 ± 25.1
		ER	−7.6 ± 6.9	12.7 ± 5.4	18.0 ± 7.6^b^	30.6 ± 3.8^B^	3.3 ± 0.3	6.6 ± 0.7	359.5 ± 72.7	60.4 ± 9.6	0.8 ± 0.1	74.4 ± 12.4
	RFS	NR	15.4 ± 3.9^a^	28.4 ± 5.3	7.6 ± 4.6^a^	33.9 ± 7.2^A^	2.9 ± 0.5	5.4 ± 0.8	87.8 ± 14.0^e^	36.5 ± 7.6	0.7 ± 0.1	60.4 ± 18.6
		ER	6.7 ± 4.1^a^^^,^^b^^	15.3 ± 3.8	14.6 ± 6.3^a^	29.8 ± 4.6	2.7 ± 0.2	5.8 ± 0.8	253.4 ± 91.7^e^	44.3 ± 7.2	0.7 ± 0.1	68.2 ± 8.6
M	FFS	NR	−12.1 ± 5.8	25.1 ± 5.3	22.0 ± 8.3^c^	47.2 ± 7.8	3.8 ± 0.7	6.9 ± 1.2	129.4 ± 36.7	58.7 ± 13.4	0.9 ± 0.2	77.7 ± 24.4
		ER	−6.9 ± 6.1	16.1 ± 5.9	12.7 ± 6.5^b^^,^c^^,^^d^^	28.9 ± 3.7^B^	3.4 ± 0.3	7.2 ± 0.7	319.7 ± 55.3	65.3 ± 7.9	0.9 ± 0.1	81.9 ± 15.4
	RFS	NR	12.9 ± 4.5^a^	30.2 ± 4.9	16.0 ± 5.2^a^^,^c^^	44.7 ± 8.8^C^	3.1 ± 0.6	5.7 ± 1.0	139.1 ± 68.0^f^	43.7 ± 10.3	0.7 ± 0.1	64.6 ± 22.5
		ER	5.1 ± 5.7^a^^,^b^^	17.5 ± 6.0	7.8 ± 4.7^a^^,^c^^,^^d^^	25.3 ± 3.9^B^	2.9 ± 0.2	6.1 ± 0.6	362.3 ± 91.4^f^	50.4 ± 8.1	0.7 ± 0.1	69.9 ± 13.2
*p*-value (*η*^2^)	RL		0.018 (0.068)	<0.001 (0.579)	0.132 (0.028)	<0.001 (0.572)	0.005 (0.096)	0.252 (0.016)	<0.001 (0.734)	0.006 (0.092)	0.028 (0.059)	0.392 (0.009)
	FW		0.276 (0.015)	0.087 (0.036)	0.897 (0.0002)	0.199 (0.021)	0.160 (0.025)	0.083 (0.037)	0.028 (0.059)	0.026 (0.061)	0.096 (0.034)	0.415 (0.008)
	FSP		<0.001 (0.718)	0.006 (0.091)	<0.001 (0.249)	0.002 (0.118)	<0.001 (0.349)	<0.001 (0.326)	0.089 (0.036)	<0.001 (0.456)	<0.001 (0.384)	0.002 (0.109)
	RL*FW		0.460 (0.007)	0.416 (0.008)	<0.001 (0.163)	<0.001 (0.141)	0.969 (0.00002)	0.380 (0.010)	0.767 (0.001)	0.700 (0.002)	0.453 (0.007)	0.722 (0.002)
	RL*FSP		<0.001 (0.170)	0.304 (0.013)	0.024 (0.062)	0.102 (0.033)	0.655 (0.003)	0.272 (0.015)	0.543 (0.005)	0.494 (0.006)	0.194 (0.021)	0.415 (0.008)
	FW*FSP		0.639 (0.003)	0.946 (0.00006)	0.356 (0.011)	0.289 (0.014)	0.670 (0.002)	0.992 (0.000001)	<0.001 (0.142)	0.345 (0.011)	0.745 (0.001)	0.947 (0.00006)
	RL*FW*FSP		0.665 (0.002)	0.550 (0.004)	0.151 (0.026)	0.041 (0.051)	0.789 (0.001)	0.342 (0.011)	0.073 (0.040)	0.506 (0.006)	0.147 (0.026)	0.505 (0.006)

C, conventional shoes; M, minimalist shoes; FFS, forefoot striking; RFS, rearfoot striking; NR, novice runners; ER, experienced runners; RL, running level; FW, footwear; FSP, foot strike patterns; *θ*_contact_, foot strike angle; *θ*_DF-peak_, peak dorsiflexion angle; *θ*_PF-peak_, peak plantar flexion angle; *θ*_ROM_, range of motion; *M*_PF-peak_, peak plantar flexion moment; AT, Achilles tendon; ILR, instantaneous loading rate; ALR, average loading rate.

^a^ Significant difference between FFS and RFS at the same running level, *p* < 0.05.

^b^ Significant difference between NR and ER in the same foot strike pattern, *p* < 0.05.

^c^ Significant difference between C and M at the same running level, *p* < 0.05.

^d^ Significant difference between NR and ER in the same footwear, *p* < 0.05.

^e^ Significant difference between FFS and RFS in the same footwear, *p* < 0.05.

^f^ Significant difference between C and M in the same foot strike pattern, *p* < 0.05.

^A^ Significant difference between FFS and RFS at the same running level and footwear, *p* < 0.05.

^B^ Significant difference between NR and ER for the same footwear and foot strike patterns, *p* < 0.05.

^C^ Significant difference between C and M at the same running level and for foot strike patterns, *p* < 0.05.

**Figure 5:**
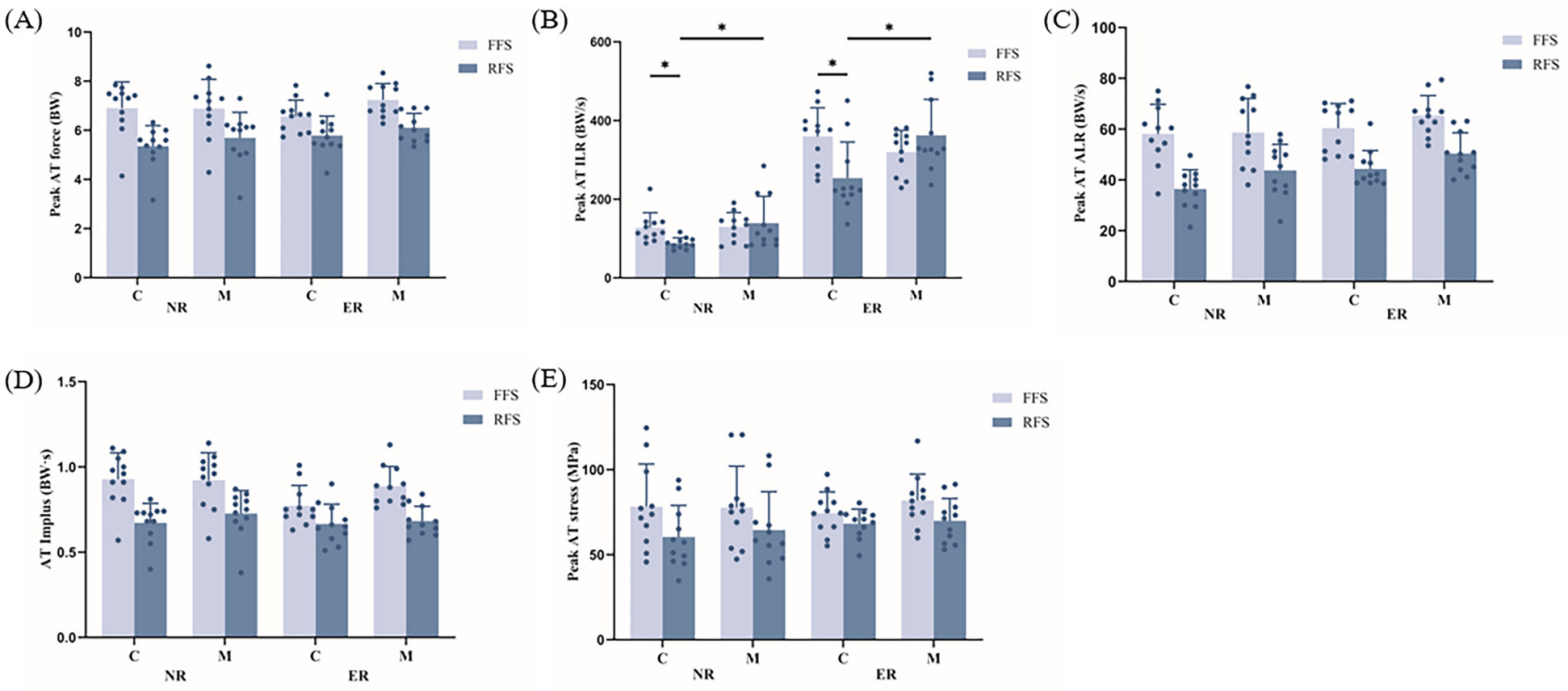
Effects of different running level, footwear and foot strike patterns on peak Achilles tendon force (AT, **A**), peak AT instantaneous loading rate (ILR, **B**), peak AT average loading rate (ALR, **C**), AT impulse **(D)**, and peak AT stress **(E)**, *p < 0.05. C, conventional shoes; M, minimalist shoes; FFS, forefoot striking; RFS, rearfoot striking; NR, novice runners; ER, experienced runners.

A significant interaction effect between footwear and foot strike patterns was observed for the AT peak ILR (*p* < 0.05, Figure 5B). *Post hoc* analyses revealed that the AT peak ILR was significantly lower for conventional shoes than for minimalist shoes during RFS and was greater for FFS in conventional shoes than for RFS. A significant main effect of the foot strike pattern was shown on the AT peak force (*p* < 0.05, Figure 5A). The results indicated that the AT peak force was significantly greater when running with FFS than with RFS. Running levels, footwear and foot strike patterns had significant main effects on the AT peak ALR (*p* < 0.05, Figure 5C). In particular, the AT peak ALR was significantly lower in novice runners, in conventional shoes and during RFS than in experienced runners, minimalist shoes and FFS. Running levels and foot strike patterns had significant main effects on AT impulses (*p* < 0.05, Figure 5D). The AT impulse was also significantly greater in novice runners and FFS than in experienced runners and RFS. The running level had a significant effect on the AT peak stress (*p* < 0.05, Figure 5E). The results indicated that the AT peak stress significantly increased when the FFS was run compared with the RFS.

## 4 Discussion

The purpose of this study was to investigate the differences in the loading characteristics of the PFJ and AT between novice and experienced runners across different footwear and foot strike patterns. The results showed that consistent with our hypotheses, novice runners had significantly greater PFCF and AT impulses than experienced runners, regardless of footwear or foot strike patterns. Moreover, we observed that adopting an FFS pattern, irrespective of running experience, could reduce PFJ loading (e.g., lower PFCF and PFJS) while increasing AT loading (e.g., higher peak AT force, impulse, ALR, and stress).

A previous study reported that novice runners were significantly more likely to experience running injuries and that the main injury sites were concentrated in the knee (30.5%) and lower leg (17.8%) (5). This study revealed that novice runners had a significant increase in PFCF of 27.8% compared with experienced runners, which was consistent with our findings. Our results revealed that novice runners had an increase in PFCF compared to experienced runners, regardless of foot strike patterns and footwear conditions. The increased PFCF observed in novice runners may be related to their greater knee flexion angle during the running stance phase, which led to greater loads on the PFJ, suggesting a significantly greater risk of patellofemoral injuries (11). In this study, we also confirmed that novice runners had a significant increase in the knee flexion angle of 37.0% compared with experienced runners. This finding was in line with previous studies by Quan et al. (18) who reported that novice runners had greater knee flexion angles than experienced runners did. This finding may be explained by the sub optimal running mechanics of novice runners, leading to increased loads on the musculoskeletal tissues, especially on the tibia and the knee joint. An increased ROM at the knee could further elevate running-related injury risks, because the surrounding soft tissues, such as the quadriceps muscle-tendon unit, were subjected to greater stretching forces (18).

This study not only assessed the risk of PFJ injury in novice runners but also considered the entire lower extremity as a kinetic chain, including the risk of AT injury. The results showed that novice runners had a significant increase in AT impulse (about 3% per step) compared to experienced runners, indicating a higher risk of AT (35). Interestingly, the ALR of the AT in novice runners was significantly lower than that in experienced runners, which seemed to contradict its greater AT force impulse. In fact, novice runners had significantly longer contact time than experienced runners and had greater step frequency and shorter step length, resulting in a running gait that appeared more “grounded”. Given that novice runners contacted for a longer period per stride and took more steps, the AT forces accumulated more over time, resulting in a greater AT impulse. Impulse, defined as the time-integrated ground reaction force, was indeed greater in experienced runners because their higher weekly mileage subjects the AT to larger cumulative loads. However, the risk of AT injury is modulated by a constellation of factors, including peak tendon force, loading rate, running kinematics, and individual morphological characteristics, rather than impulse magnitude alone (36). Compared to experienced runners, novice runners had a 76.1% greater ankle dorsiflexion angle and a 37.0% greater knee flexion angle. These greater angles altered the length-tension relationship of the gastrocnemius muscle. Greater ankle dorsiflexion and knee flexion angles increase the external moment arm at the knee and ankle joints and decrease the effective mechanical advantage, thus increasing AT loading in novice runners and leading to a greater risk of AT injury (37). Therefore, the present study found that novice runners exhibited less refined mechanics, specifically, increased knee and ankle flexion angles, longer contact times, and greater gait variability. These patterns elevate both instantaneous Achilles loading and per step impulse, thereby heightening injury risk despite lower overall mileage. By contrast, prolonged training in experienced runners promoted beneficial adaptations, including greater tendon stiffness and more efficient elastic energy storage and return, enabling them to withstand higher absolute loads with comparatively lower risk (38).

The results of this study align with previous research on the immediate effects of changing foot strike patterns. The study found that PFJS was significantly lower during immediate FFS than RFS, regardless of running level or footwear, indicating that even habitual RFS runners experienced a similar reduction in PFJ load when the immediate transition to FFS. For example, Kulmala et al. (39) found that habitual FFS runners had lower PFJS and PFCF than RFS runners, mainly due to a smaller knee extension moment during FFS. While there was no significant difference in PFCA between foot strike patterns in this study, FFS resulted in significantly lower PFJ stress, reducing knee load. This finding supports Xu et al. (40), who noted that FFS reduced knee extension moments, while RFS increased knee energy absorption, raising PFJS.

The study found that peak AT forces during running ranged from 5.3 to 7.2 BW, similar to musculoskeletal modelling results (5–7 BW) (41, 42). Additionally, peak plantar flexion moment and peak AT force increased by 22.4% and 20.0%, respectively, when transitioning from RFS to FFS. This aligns with Kulmala et al. (39), who reported 24% greater AT force in FFS runners. The increase in AT force was due to greater plantar flexion moments during FFS, which changed calf muscle and AT activation patterns, leading to higher AT loads. As the AT cross-sectional area did not change rapidly, the immediate transition to FFS also increased AT stress, with an 18.7% rise compared to RFS. Furthermore, the AT impulse was significantly higher with FFS, consistent with greater AT loading. This was supported by Rice and Patel (10) who found higher AT impulses in habitual RFS runners using minimalist shoes with FFS. Sudden shifts from RFS to FFS can increase AT loading, and while gradual changes may be beneficial, abrupt transitions often lead to AT injuries (25, 26).

Most previous studies have looked at the PFJ or AT separately, but the lower limb should be viewed as a complete kinetic chain. This study analyzed the combined effects of footwear and foot strike patterns on both PFJ and AT loading, focusing on novice runners to provide practical recommendations. The higher PFJ and AT loads in novice runners were linked to poor running technique and posture, leading to excessive knee flexion, increased PFCF, and greater ankle plantar flexion (18, 37, 43). Additionally, their shorter step length and faster step frequency, indicating insufficient propulsion, further increased AT impulses and the risk of injury. Therefore, novice runners should focus on preventing injuries to both the PFJ and AT. The choice of footwear and foot strike pattern is important for managing loading on these joints. Regardless of the running level, special attention should be given to changes in foot strike patterns while considering different footwear. While FFS can reduce PFJS (39), it shifts the load to the AT (32), so novice runners, particularly those with knee pain, should gradually adapt to FFS by reducing knee flexion and strengthening lower limb muscles to minimize impact on both the PFJ and AT (26). Moreover, runners should improve neuromuscular control of knee and ankle joint flexion, optimize step frequency and stride length, and elect footwear that matches their biomechanical needs. Although these recommendations are particularly relevant for novices, several are equally pertinent for experienced runners, such as adopting a foot-strike pattern that lowers patellofemoral-joint loading or strengthening the lower limb muscles. These strategies are not only effective in reducing the risk of injury to runners but also in optimising performance.

This study has certain limitations. First, the experiment was conducted in a lab, which may differ biomechanically from outdoor running in a natural environment. Second, only male participants were included due to higher biomechanical loads in males during daily activities (44). The impact of running levels, foot strike patterns, and footwear on female runners' PFJ and AT is unclear, so future studies should address gender differences. Lastly, this study focused on the immediate effects of changing foot strike patterns and footwear. Future research should explore the long-term effects of these changes, combined with training, on PFJ and AT loading.

## 5 Conclusion

The findings of this study indicated that novice runners experienced significantly greater PFJ and AT loading during running than experienced runners did. Regardless of footwear conditions and foot strike patterns, novice runners showed greater angular changes in the knee and ankle joints, leading to an increased PFCF and AT impulse. Furthermore, regardless of running experience, the AT impulse, force and stress significantly increased, whereas the PFJS significantly decreased when an FFS pattern was adopted immediately. Transitioning to FFS may reduce PFJ loading, particularly for novice runners, but it simultaneously increases the risk of injuries such as Achilles tendinopathy. Therefore, novice runners should gradually adapt their foot strike patterns based on the load-bearing capacity of specific joints, thereby minimising the risk of joint-related injuries.

## Data Availability

The raw data supporting the conclusions of this article will be made available by the authors, without undue reservation.
